# Viral Infectivity in Patients Undergoing Tracheotomy With COVID-19: A Preliminary Study

**DOI:** 10.1177/01945998211004255

**Published:** 2021-12

**Authors:** Manish M. George, Charlotte J. McIntyre, Jie Zhou, Ruthiran Kugathasan, Dora C. Amos, Ivan J. Dillon, Wendy S. Barclay, Neil S. Tolley

**Affiliations:** 1 Imperial College London, London, UK; 2 Imperial College NHS Healthcare Trust, London, UK

**Keywords:** tracheotomy, COVID-19, SARS, SARS-CoV-2, infectivity, antibodies, PCR

## Abstract

**Objective:**

To establish the presence of live virus and its association with polymerase chain reaction (PCR) positivity and antibody status in patients with COVID-19 undergoing tracheotomy.

**Study Design:**

Prospective observational study.

**Setting:**

Single institution across 3 hospital sites during the first wave of the COVID-19 pandemic.

**Methods:**

Patients who were intubated for respiratory wean tracheotomy underwent SARS-CoV-2 PCR nasal, throat, and endotracheal tube swabs at the time of the procedure. These were assessed via quantitative real-time reverse transcription PCR. The tracheal tissue excised during the tracheotomy was cultured for SARS-CoV-2 with Vero E6 and Caco2 cells. Serum was assessed for antibody titers against SARS-CoV-2 via neutralization assays.

**Results:**

Thirty-seven patients were included in this study. The mean number of days intubated prior to undergoing surgical tracheotomy was 27.8. At the time of the surgical tracheotomy, PCR swab testing yielded 8 positive results, but none of the 35 individuals who underwent tissue culture were positive for SARS-CoV-2. All 18 patients who had serum sampling demonstrated neutralization antibodies, with a minimum titer of 1:80.

**Conclusion:**

In our series, irrespective of positive PCR swab, the likelihood of infectivity during tracheotomy remains low given negative tracheal tissue cultures. While our results do not undermine national and international guidance on tracheotomy after day 10 of intubation, given the length of time to procedure in our data, infectivity at 10 days cannot be excluded. We do however suggest that a preoperative negative PCR swab not be a prerequisite and that antibody titer levels may serve as a useful adjunct for assessment of infectivity.

Severe acute respiratory syndrome coronavirus 2 (SARS-CoV-2) developed into a global pandemic in early 2020. Total infected numbers continue to grow worldwide as we encounter second and further waves. Despite having overall lower mortality than the SARS outbreak in 2003, SARS-CoV-2 is considerably more infectious, with a median 5-day incubation period and asymptomatic spread.^1,2^

As expected, medical professionals are at particularly high risk due to patient proximity, with Wuhan seeing health care workers represent 3.8% of infected patients and Italy noting up to 15%.^3,4^ SARS-CoV-2 particles are mainly transmitted via droplets of approximately 5 to 10 µm. Aerosolization, however, can reduce this size to <0.5 µm with these microdroplets remaining airborne for up to 3 hours.^5^ A large number of aerosol-generating procedures (AGPs) have been identified, and these include and are not limited to intubation, airway suction, endoscopy of the upper aerodigestive tract, skull base drilling, and tracheotomy.^6,7^ Staff present during these AGPs are at especially high risk of infection.^8^ Further studies demonstrated that otolaryngologists are among the most exposed to SARS-CoV-2, and many have advocated special protective measures to minimize infection risk.^9,10^

While the majority of patients who are infected remain asymptomatic or develop only mild symptoms, up to 20% need respiratory support in an intensive care unit (ICU).^11^ Many of these patients require prolonged support, and to limit risks of lip and oropharyngeal necrosis alongside laryngeal and subglottic stenosis, tracheotomy continues to play a key role in management.^12^ Tracheotomy allows earlier weaning from the ventilator, which not only reduces complications associated with prolonged intubation, but also frees up limited resources for other patients who may require ventilation.

There has been particular unease within the otolaryngology community with performing tracheotomies, a known AGP. A systematic review demonstrated an increased risk in contracting SARS during the 2003 outbreak for those conducting tracheotomy, at an odds ratio of 4.15.^13^ These concerns have been heightened with reported respiratory personal protective equipment shortages and variable access to powered air-purifying respirators. Furthermore, exposure of viral load may have a dose-dependent association to the severity of disease, with fears that AGPs generate high volumes of inhalable infectious virus particulates. A study in China found a clear correlation between viral load from nasopharyngeal swabs and symptom severity.^14^ While this study does not assess initial exposure dose, higher infectious viral dose has been associated with worsened severity of disease in influenza.^15^ Supporting this hypothesis are anecdotal reports of high rates of severe infection in ear, nose, and throat and ophthalmology staff due to patient airway proximity on examination.^9,10^

It remains unclear at what stage of disease surgical tracheotomy should be undertaken, balancing patient benefit and risk to health care workers. Furthermore, the published guidance is varied on minimum length of time from intubation and the requirement for negative polymerase chain reaction (PCR) swabs pretracheotomy.^16-19^

Perhaps most notably, true infectivity does not necessarily equate to PCR swab positivity. Viral RNA fragments can remain in circulation or at the mucosal surface for several days, if not weeks, after viable virus particles have been cleared by the immune system.^20-22^ A more reliable investigation to establish the presence of viable viral particles includes culture in cell lines.^20,22,23^ Wolfel et al^24^ studied the presence of live SARS-CoV-2 in patients who were COVID-19 positive. Virologic analysis, including culture of 9 patients, was undertaken isolating live virus from the throat and lungs of all patients. While some recent studies suggest low to negligible viable viral particles by day 10, there are little to no specific data on infectivity during COVID-19 tracheotomy.^23,24^

Tracheal windows are excised during surgical tracheotomies, providing tissue that can be opportunistically used for culture. This can demonstrate the presence or absence of live virus. The trachea is necessarily the site that surgeons encounter during this procedure and is therefore among the most appropriate for surrogate measure of infectivity and exposure.

The purpose of this study is to determine (1) the infectivity of patients with COVID-19 undergoing surgical tracheotomy by using tracheal tissue culture of live SARS-CoV-2, (2) the presence of serum antibody titers against SARS-CoV-2, and (3) the relationship of SARS-CoV-2 PCR swabs to isolated live virus.

## Methods

### Data Collection

#### Sample Collection

Data collection took place at a single institution across 3 hospitals during the first wave of the pandemic, between April 21 and May 29, 2020. Eligible patients were defined as those who were positive for COVID-19 and were undergoing ventilation in the ICU and tracheotomy for respiratory wean.

The patients’ demographics and dates of symptom onset, intubation, and tracheotomy were recorded. All patients underwent SARS-CoV-2 PCR swabs of the nasopharynx intraoperatively. Some underwent oropharynx and endotracheal tube swabs. At the time of surgery, the tracheal window, which was excised as part of the standard tracheotomy protocol, was preserved and sent for viral culture. Additionally, blood serum sample was taken for SARS-CoV-2 antibody titers.

For each participant, the nasal swab, throat swab, and endotracheal tube swab were collected with flocked swabs and preserved in universal transport medium (catalog 305C; Copan UTM System) or in Amies (catalog 480C; ESwab Collection System). Tracheal tissue windows were preserved in saline. All samples were transported on ice to a containment level 3 laboratory within 24 hours. Tracheal tissue samples were homogenized with the TissueLyser LT (Qiagen); the supernatants were used for analysis after centrifuging. Blood serum was tested for SARS-CoV-2 antibody titer in neutralization assays with wild type and pseudotype virus.

#### Detection and Quantification of SARS-CoV-2 Viral RNA Genome

Viral RNA detection and absolute quantification were performed with quantitative real-time reverse transcription PCR (RT-qPCR). Samples were extracted from 140 µL with the QIAamp Viral RNA Mini Kit according to the manufacturer’s instructions (Qiagen). Negative controls (water) were extracted and included in the PCR assays. SARS-CoV-2 viral RNA was detected with AgPath-ID One-Step RT-PCR Reagents (Life Technologies) with specific primers and probes targeting the E gene (envelop).^25^ All samples were run in duplicate.

#### Virus Culture

Vero E6 cells (African green monkey kidney) and Caco2 cells (human colon carcinoma) were used to culture virus from samples. The cells were cultured in DMEM supplemented with heat-inactivated fetal bovine serum (10%) and penicillin-streptomycin (10,000 IU/mL and 10,000 µg/mL). For propagation, 200 µL of samples was added to 24-well plates. After 5 to 7 days, cell supernatants were collected, and RT-qPCR to detect SARS-CoV-2 was performed as described. Samples with at least a 1-log increase in copy numbers for the E gene (reduced cycle threshold [CT] values relative to the original samples) after propagation in cells were considered positive by viral culture.

#### Pseudotype Neutralization Assay Method

Patient serum was initially diluted 1:10 and then serially diluted 1:5. SARS-CoV-2–pseudotyped virus was added to each serum dilution and incubated at 37 °C for 1 hour. The serum-pseudotyped virus mixture was added to HEK-293T cells stably transfected to express ACE2 and incubated at 37 °C for 48 hours. The Bright-Glo Lucifearase Assay System (Promega) was used to lyse cells and produce the luciferase readout, which was measured with a FLUOstar Omega plate reader (BMG Labtech). Relative luminescence units were normalized to pseudovirus and media-alone readouts. IC_50_ values were calculated by nonlinear regression (Prism; GraphPad).

#### Wild Type Virus Neutralization Assay Method

The ability of patient serum to neutralize wild type SARS-CoV-2 virus was assessed by neutralization assay on Vero E6 cells. Heat-inactivated sera were serially diluted in assay diluent consisting of DMEM (Gibco, Thermo Fisher Scientific) with 1% penicillin-streptomycin (Thermo Fisher Scientific) and 0.3% bovine serum albumin fraction V (Thermo Fisher Scientific). Serum dilutions were incubated with 100 TCID_50_ per well of virus in assay diluent for 1 hour at room temperature and transferred to 96-well plates preseeded with Vero E6 cells. Serum dilutions were performed in duplicate. Plates were incubated at 37 °C, 5% CO_2_, for 4 days before an equal volume of 2× crystal violet stain was added to wells for 1 hour. Plates were washed; wells were scored for cytopathic effect; and a neutralization titer was calculated as the reciprocal of the highest serum dilution at which full virus neutralization occurred.

### Statistical Analysis

The Shapiro-Wilk test for normality was used on all data sets and subsets of data. Where the data were parametric, they were presented as mean and SD. Pearson rank correlation was used to assess for correlation between variables. Where the data were nonparametric, the median and interquartile range were presented. Spearman rank correlation was used to assess for correlation between variables.

### Ethics Committee Approval

This project has approval from the Health Research Authority (project 283590; Integrated Research Application System).

## Results

### Demographics

A total of 40 participants underwent surgical tracheotomy during the data collection period. Three were excluded due to incomplete data. The mean ± SD age of the participants was 56 ± 8 years. The male:female ratio was 2.4:1 (24 males and 10 females).

### Timeline

**Figure 1.**
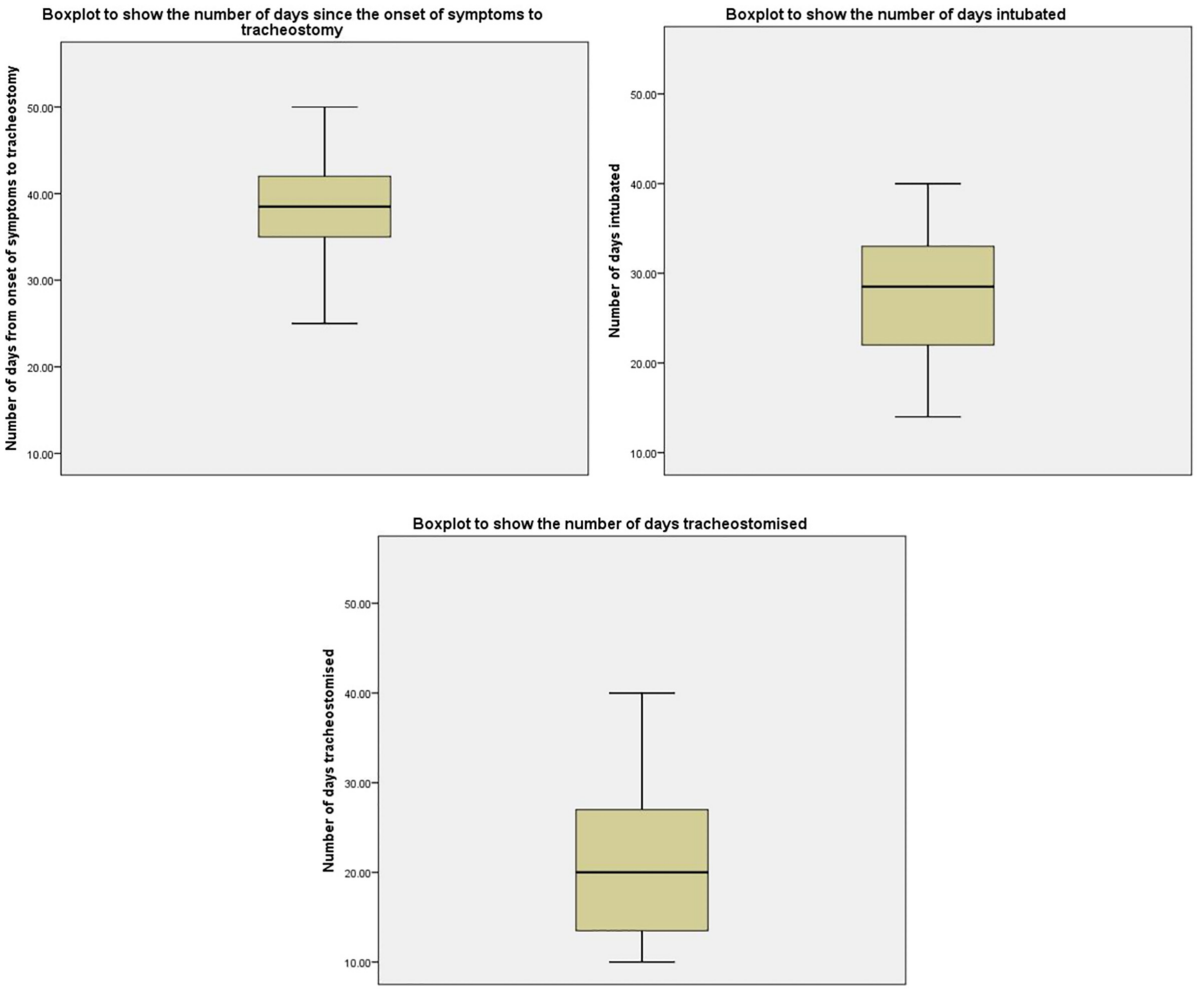
Box plots demonstrating the number of days since the onset of symptoms to tracheotomy, the number of days intubated, and the number of days tracheotomized (days with a tracheotomy tube prior to decannulation). Values in days are presented as mean (line), SD (box), and range (whiskers).

The mean number of days from the onset of symptoms to undergoing the surgical tracheotomy was 38.5 ± 6.98, with a range of 34 to 68 days. The mean number of days that the patients spent intubated prior to undergoing a surgical tracheotomy was 27.8 ± 7.18, with a range of 14 to 40 days. At the time of article submission, 32 patients had undergone decannulation, and of these, the mean length of time spent with a tracheotomy tube was 30 ± 8.71 days, with a range of 10 to 40 days (**Table 1**, **Figure 1**).

**Table 1.** Tracheotomy Timeline.

	No. of days		
	Mean	SD	Range
Tracheotomy			
Symptom onset to	38.5	6.98	34-68
Intubated prior to	27.8	7.18	14-40
Time with an endotracheal tube	30.0	8.71	10-40

### CT and Quantity Reports

**Figure 2.**
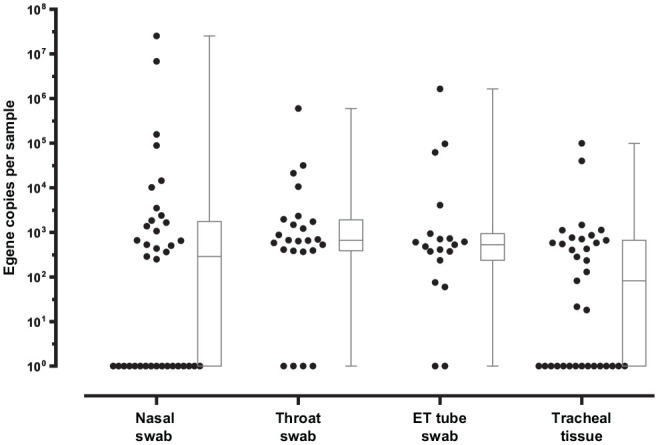
Box plots demonstrating quantity (virus copies) per sample collected in nasal swab (n = 37), throat swab (n = 24), endotracheal (ET) tube swab (n = 19), and tracheal tissue (n = 35), as quantified by quantitative real-time reverse transcription polymerase chain reaction in all samples. Values in virus copies are presented as mean (line), SD (box), and range (whiskers).

For each participant, the nasal swab, throat swab, endotracheal tube swab (tube swab), and tracheal tissue sample underwent analysis. The CT and quantity were reported. Quantity calculated per swab and swab type is illustrated in **Figure 2**. The CT value refers to the number of cycles required for the fluorescent signal of the target nucleic acid to reach the threshold for detection. Typically, a value ≤29 indicates a strong positive reaction; 29 < CT ≤ 36, a weak positive reaction; and >36, a negative reaction. Of 37 patients, 8 had at least 1 positive swab result on RT-qPCR. The distribution of positive and negative results for each type of sample is shown in **Table 2**. **Table 3** shows the distribution of CT values among positive swabs in our data set.

**Table 2.** Number of Strongly and Weakly Positive, Negative, and Unavailable Swabs.

Type of sample	Strongly positive (CT ≤29)	Weakly positive (29 < CT ≤36)	Negative (CT >36)	Not available
Nasal swab	2	6	29	0
Throat swab	1	4	19	13
Endotracheal tube swab	1	3	15	18
Tracheal tissue	0	2	33	2

Abbreviation: CT, cycle threshold.

**Table 3.** Median, IQR, and Range of Cycle Threshold Values for the Positive Samples.

Cycle threshold	Median	IQR	Range
Nasal swab	33.7	13.1	22.4-39.1
Throat swab	37.8	6.20	27.5-38.8
Endotracheal tube swab	37.8	2.76	34.7-41.1
Tracheal tissue	38.4	2.76	30.5-43.0

Abbreviation: IQR, interquartile range.

**Figure 3.**
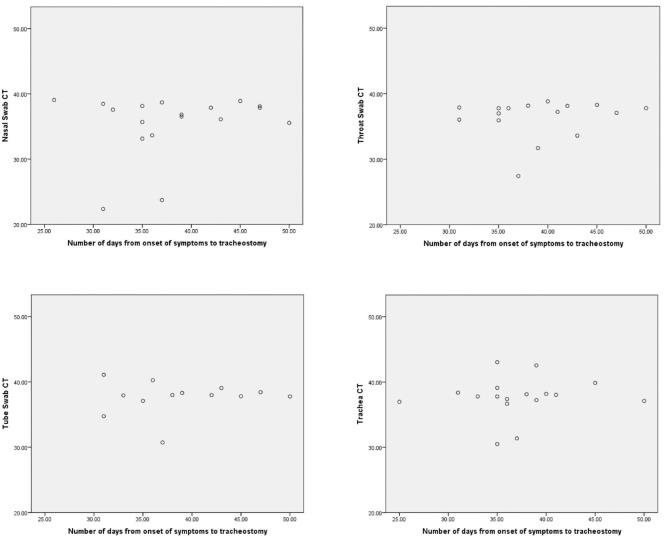
Scatter plots show the number of days from the onset of symptoms to tracheotomy vs the sample cycle threshold (CT) values.

**Figure 4.**
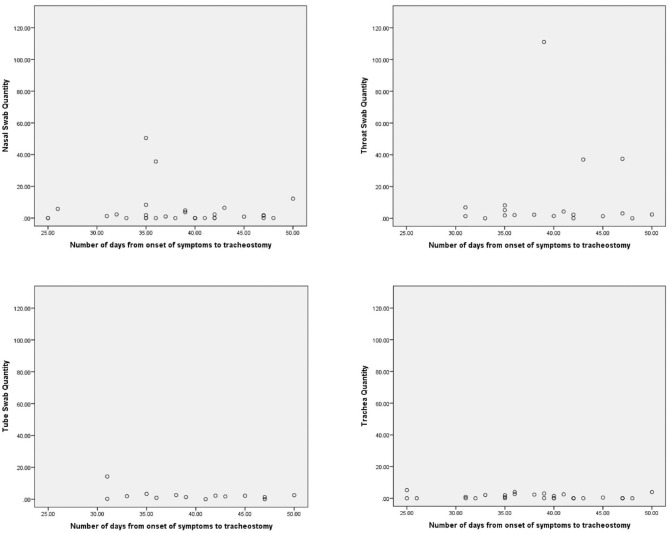
Scatter plots show the number of days from the onset of symptoms to tracheotomy vs the sample quantity values.

Pearson and Spearman rho rank correlation was used to assess correlation between the total days since symptom onset and the CT value and quantity (parametric and nonparametric data sets, respectively). All data underwent a Shapiro-Wilk test to check for normality. None of the CT values or quantities were significantly correlated with total days since symptom onset (**Table 4**, **Figures 3** and **4**).

**Table 4.** Correlation Between Cycle Threshold Value or Quantity and Number of Days Since Symptom Onset.

	Correlation coefficient		
Type of sample	Pearson	Spearman rho	*P* value
Cycle threshold			
Nasal swab	0.210		.403
Throat swab		0.270	.311
Endotracheal tube swab	−0.024		.938
Trachea		0.176	.515
Quantity			
Nasal swab	−0.095		.604
Throat swab		−0.189	.483
Tube swab		−0.94	.761
Trachea	−0.288		.280

### Tracheal Tissue Culture

Thirty-five samples of tracheal tissue underwent culture. In this study of 35 patients, live virus was not grown on tracheal tissue after 34 days following the onset of symptoms.

### Antibody Testing

Eighteen participants had blood serum tested for antibodies against the spike protein. Of these, all 18 test results were positive for antibodies. A Shapiro-Wilk test demonstrated that the antibody titer results were not normally distributed. The median antibody titer was 1:640, with an interquartile range of 800. The range was 1:80 to 1:1120. We did not find any significant correlation between the antibody titer and the number of days since the onset of symptoms. Using Spearman rank correlation, we calculated the correlation coefficient to be –0.257, which was not significant (*P* = .304). A significant correlation was also not found after removal of the outlier antibody titer (1:2560).

## Discussion

Tracheotomies are an important surgical procedure conducted by ear, nose, and throat surgeons, alongside other surgical specialties. In the semielective setting as part of the respiratory wean, tracheotomies are of morbidity-reducing and prognostic value, and in general, an earlier procedure is supported by the literature.^26,27^

During the COVID-19 pandemic, however, special considerations have been given to minimize risk to staff, often resulting in delays of tracheotomy, especially against the background of increased infectivity demonstrated with other coronaviruses.^8,28^ No specific data on risk of contracting COVID-19 during a tracheotomy have been described to date.

Our study is the first of its kind, using superior methods of tissue viral culture along cell lines to investigate the presence of any live SARS-CoV-2 on tracheal tissue in patients with COVID-19 at the time of undergoing surgical tracheotomy. We provide quantitative results demonstrating that at the planned stage of tracheotomy, several patients have detectable viral RNA (8 of 37), some with high numbers of genome copies. Most important, however, our results show no live SARS-CoV-2 on tracheal tissue in all patients who underwent tissue culture. Of note, no staff members involved in the surgical procedure or the tracheotomy care of these patients contracted COVID-19 during this period, mirroring results from the national tracheotomy audit review of 564 cases in COVIDTrach.^28^ While the sensitivity of cell cultures is dependent on factors ranging from cell type to culture medium and effective sampling, culture remains the gold standard in assessing infectivity.^29,30^

It has been demonstrated that PCR swab positivity, or “viral shedding,” after the early course of disease, does not correlate with virus infectivity.^21,31^ An early small series of specimens demonstrated no live virus isolation after day 8 of illness despite high viral RNA loads.^24^ This fall in infectivity is especially marked in the presence of detectable antiviral antibodies, and a serum-neutralizing antibody titer has been associated with noninfectious SARS-CoV-2.^32^ All 18 tested patients demonstrated antibodies to SARS-CoV-2, supporting these studies. Furthermore, our minimum detected antibody titer of 1:80 correlated well with a recent study of 129 patients which demonstrated no culturable virus with detected antibody titers of 1:80 and above.^32^ For this reason, there may be value in considering antibody serologic testing as an adjunct or even a substitute to a SARS-CoV-2 PCR swab. Guidance from the National Tracheotomy Safety Project has mentioned that a “negative” SARS-CoV-2 RNA PCR result is not necessary prior to undertaking tracheotomy, and our results concur.^16^

Our study has some perceived limitations. First, we recognize that culture from tracheal tissue alone may not provide a comprehensive assessment of infectivity across all potential sites of infection. We also note in our results, a relatively lower PCR RNA volume in the trachea when compared with the nasopharynx. However, other studies have shown that in most patients, lower respiratory tract samples remain PCR positive for up to 39 days after samples from the upper respiratory tract become negative.^33,34^ More comprehensive studies demonstrate that upper respiratory tract specimens tend to give poorer diagnostic yield than lower respiratory specimens.^35^ This suggests that the ideal location for culture sample in severe and late illness may indeed be the lower respiratory tract rather than nasopharynx. In addition, a tissue biopsy of the nose or nasopharynx may provide further information for culture; however, this procedure may also harm, especially in an anticoagulated patient. In the absence of consent from a patient who is sedated, this was deemed unjustified. Tracheal windows however are ordinarily discarded posttracheotomy. We believe that tracheal tissue works as a sufficient surrogate for the assessment of infectivity, especially given its specificity to the tracheobronchial tree, the main source of aerosol. Furthermore, neutralizing antibodies were detected in all tested patients, including those with positive viral RNA on PCR. Given the strong associations described between neutralizing antibodies and lack of infectivity, it is unlikely that any difference in culture success would be observed.^32^

Second, while many guidelines recommend tracheotomy after 10 days to 2 weeks from intubation, the mean length in our series was 27.8 days. The national tracheotomy audit COVIDTrach also saw delays beyond prepandemic protocols.^28^ Tracheotomies in our study were undertaken within 4 days of request from the ICU team, suggesting that postponed request was the underlying reason for a delay in performing the tracheotomy. It is likely that the initial lack of knowledge regarding infectivity and patient recovery in poorly compliant lungs resulted in delays in the decision for tracheotomy. While this limitation may restrict applicability to earlier tracheotomies in patients with COVID-19, we note that in a subgroup analysis of earlier procedures, at days 14 to 20 postintubation, all tested patients had neutralizing antibodies present in sera, and all demonstrated no live virus cultured from tissue.

Third, our study size is relatively small. It is worth remembering that in patients with severe disease—by definition, those undergoing tracheotomy—the reported viral RNA load is significantly higher and decreases more gradually.^36,37^ While our numbers provide reassurances, we recognize that our patient cohort is not sufficiently large, and we caution against the assumption that there is no infectivity in any patient with COVID-19 who is eligible for tracheotomy. Importantly, we are not advocating for reduced personal protective equipment during tracheotomy, and ultimately the decision regarding the degree of protection should be taken at the local level, ensuring that the multidisciplinary team feels appropriately protected. In our series, no staff member in theater contracted SARS-CoV-2 within 2 weeks of tracheotomy.

The results of this study have implications in the delivery of clinical care to patients with COVID-19 in ICUs. Our series does not demonstrate any live SARS-CoV-2 on the tracheal tissue samples at the time of the surgical tracheotomy irrespective of PCR positivity. Our results do not undermine national and international guidance on tracheotomy after day 10 of mechanical ventilation.^18,38^ However, given the length of time to procedure in our data set, infectivity at 10 days cannot be excluded. The results, however, reinforce the poor positive predictive value of viral RNA PCR in infectivity later in the illness course; therefore, a negative swab may not be necessary. We recognize that antibody titer levels may serve as a useful adjunct for assessing infectivity. Importantly, further studies at an earlier time point would assess infectivity more comprehensively.

Research into COVID-19/SARS-CoV-2 is an evolving situation where policy should be guided by the best available evidence. While in our results, time from symptom onset to performing surgical tracheotomy was longer than pre–COVID-19 timings, it should provide some reassurance to the health care providers involved in tracheotomy surgery and posttracheotomy care.
